# Characterization of PA-N terminal domain of Influenza A polymerase reveals sequence specific RNA cleavage

**DOI:** 10.1093/nar/gkt603

**Published:** 2013-07-11

**Authors:** Kausiki Datta, Andrea Wolkerstorfer, Oliver H. J. Szolar, Stephen Cusack, Klaus Klumpp

**Affiliations:** ^1^Hoffmann-La Roche Inc., Virology Discovery, Nutley, NJ 07110, USA, ^2^Savira pharmaceuticals GmbH, Veterinaerplatz 1/IA, A-1210, Vienna, Austria, ^3^European Molecular Biology Laboratory, Grenoble Outstation, 6 rue Jules Horowitz, BP181, 38042 Grenoble Cedex 9, France, ^4^Unit of Virus Host Cell Interactions, University Grenoble Alpes-EMBL-CNRS, 6 rue Jules Horowitz, BP181, 38042 Grenoble Cedex 9, France and ^5^RiboScience LLC, 3901 Laguna Avenue, Palo Alto, CA 94306, USA

## Abstract

Influenza virus uses a unique cap-snatching mechanism characterized by hijacking and cleavage of host capped pre-mRNAs, resulting in short capped RNAs, which are used as primers for viral mRNA synthesis. The PA subunit of influenza polymerase carries the endonuclease activity that catalyzes the host mRNA cleavage reaction. Here, we show that PA is a sequence selective endonuclease with distinct preference to cleave at the 3′ end of a guanine (G) base in RNA. The G specificity is exhibited by the native influenza polymerase complex associated with viral ribonucleoprotein particles and is conferred by an intrinsic G specificity of the isolated PA endonuclease domain PA-Nter. In addition, RNA cleavage site choice by the full polymerase is also guided by cap binding to the PB2 subunit, from which RNA cleavage preferentially occurs at the 12th nt downstream of the cap. However, if a G residue is present in the region of 10–13 nucleotides from the cap, cleavage preferentially occurs at G. This is the first biochemical evidence of influenza polymerase PA showing intrinsic sequence selective endonuclease activity.

## INTRODUCTION

Influenza continues to impose a substantial annual burden of morbidity and mortality. Current antiviral drugs approved for the treatment of influenza virus infections are limited to two viral targets, neuraminidase and M2 ion-channel protein. The viral RNA (vRNA)-dependent RNA polymerase (RdRP) that is central to the virus lifecycle is a promising target for the development of novel influenza antiviral compounds.

The influenza virus contains a segmented RNA genome with eight negative-sense segments (1). The viral polymerase is a heterotrimer of subunits PA, PB1 and PB2 with a combined mass of ∼250 kDa. The RdRP trimer exists as part of a viral ribonucleoprotein complex (vRNP) and carries out the two distinct processes of transcription (synthesis of capped, polyadenylated mRNA) and replication (synthesis of full-length genomic RNA). Transcription involves (i) binding of the 5′ cap (m^7^GTP) of a host pre-mRNA to the PB2 subunit, (ii) cleaving of a phosphodiester bond 10–13 nt downstream of the cap by the endonuclease activity in the PA subunit and (iii) initiating synthesis of viral mRNAs at the cleaved 3′ end of the capped segment by the PB1 subunit that harbors the polymerase active site [reviewed in (2,3)]. The polymerase complex also replicates the vRNAs in a distinctly different process that involves unprimed initiation of RNA synthesis (4). It is unclear how these different processes are coordinated between the polymerase subunits and the difficulty in obtaining active trimeric influenza polymerase has hampered the characterization of this important multifunctional enzyme. Recent breakthroughs in the elucidation of the structure of cap binding (5) and PA-N terminal (PA-Nter) endonuclease (6,7) domains of the polymerase have begun to reveal some architecture and specific roles of the individual subunits and also allow structure-based anti-influenza drug development (8,9).

In this study, we have used enzymatic and spectroscopic approaches to understand the RNA cleavage characteristics of the PA-Nter domain on capped and uncapped RNA. The cleavage of host pre-mRNA by PA-Nter is essential for the transcription of viral mRNAs and is likely involved in the modulation and shut-down of host protein expression; however, the detailed mechanism underlying RNA selection, RNA binding and cleavage are not well understood. The structure of PA-Nter domain corresponding to the first 209 N-terminal amino acid residues shows that it contains a divalent metal ion-dependent endonuclease active-site consistent with the previously determined metal ion catalyzed cleavage reaction using native influenza polymerase (10). The metal-binding residues His41, Glu80, Asp108 and Glu119 and the catalytic lysine K134 are conserved amongst all influenza A and B strains (9,11). However structural information showing the mode of RNA binding to PA-Nter is currently lacking. Here, we show biochemically that the PA-Nter displays a remarkable RNA sequence selectivity of RNA cleavage. A comparative study with native influenza vRNP obtained from purified virus revealed a similar selectivity and suggests that host pre-mRNA sequence selectivity exhibited by influenza virus polymerase is conferred by the architecture of the minimal PA endonuclease domain.

## MATERIALS AND METHODS

### RNA and protein

Unlabeled and 2-aminopurine (2-AP)-labeled uncapped RNA oligonucleotides were purchased from Thermo Fisher Scientific. The 5′-triphosphorylated RNAs were purchased from Fidelity Systems (Gaithersburg, MD). Fifty-mer RNA was generated by T7 RNA polymerase mediated transcription of template DNA. Capping reactions were performed using the ScriptCap™ m^7^G Capping System purchased from CellScript (Madison, WI). RNA sequences and nomenclature are shown in Tables 1 and 2. Oligonucleotide concentrations were determined by ultraviolet (UV) spectroscopy, using extinction coefficients furnished by the manufacturer.

**Table 1.** Nomenclature and sequence for RNAs used in this study: all substrate RNAs and the endonuclease products of G20 RNA

Nomenclature	RNA sequence
uc-GC-1	5′p-AAU CUA UAA UAA UUA GCA CC-3′
c-GC-1	5′m^7^G-ppp-AAU CUA UAA UAA UUA GCA CC-3′
uc-GC-2	5′p-AAU CUA UAA UAA GCA UAU CC-3′
uc-GC-3	5′p-AAU CUA UAA GCA UUA UAU CC-3′
uc-GC-4	5′p-AAU CUA GCA UAA UUA UAU CC-3′
uc-GC-5	5′p-AAA GCA UAA UAA UUA UAU CC-3′
uc-G20	5′p-GAA UAC UCA AGC UAU GCA UC-3′
uc-CG20	5′p-AAU CUA UAA CGA UUA UAU CC-3′
uc-AU20	5′p-UAA UAA AUA AUA AAU AAA UU-3′
uc-G20-2AP	5′p-GAA UAC UCA AGC **X**AU GCA UC-3′
uc-G20-2AP-P1	5′p-GAA UAC UCA AGC **X**AU G-3′
uc-G20-2AP-P2(3′)	5′p-C**X**A UGC AUC-3′
uc-G20-P1	5′p-GAA UAC UCA AGC UAU G -3′
uc-G20-P2	5′p-GAA UAC UCA AG-3′

For c-RNA, the 5′-cap is the same for all and is shown for the first sequence only. The GC motif is underlined for each sequence. The position of 2-AP substitution is denoted as **X**.

**Table 2.** Nomenclature and sequence for all uncapped and capped RNA markers used in this study

Nomenclature	RNA sequence
uc-G20-16mer(M)	5′p-GAA UAC UCA AGC UAU G-3′
uc-G20-11mer(M)	5′p-GAA UAC UCA AG-3′
uc-G20-4mer(M)	5′p-GAA U-3′
uc-G20-3mer(M)	5′p-GAA-3′
uc-G20-2mer(M)	5′p-GA-3′
uc-GC3-11mer(M)	5′p-AAU CUA UAA GC-3′
uc-GC3-10mer(M)	5′p-AAU CUA UAA G-3′
c-G20-16mer(M)	5′m^7^G-ppp-GAA UAC UCA AGC UAU G-3′
c-G20-11mer(M)	5′m^7^G-ppp-GAA UAC UCA AG-3′
c-GC1-16mer(M)	5′m^7^G-ppp-AAU CUA UAA UAA UUA G-3′
c-GC2-13mer(M)	5′m^7^G-ppp-AAU CUA UAA UAA G-3′
c-GC3-11mer(M)	5′m^7^G-ppp-AAU CUA UAA GC-3′
c-GC3-10mer(M)	5′m^7^G-ppp-AAU CUA UAA G-3′

PA-Nter domain [residues 1-209, A/Victoria/3/1975(H3N2)] was expressed and purified as previously described, and the concentration of the protein was determined by UV spectroscopy, using ε_280_^1% ^= 8.79 (6).

RNP complexes were isolated from purified Influenza virus A/PR/8/34 (H1N1) purchased from Charles River Laboratories (Wilmington, MA), using an established protocol, and RNP concentration was determined by UV spectroscopy, using OD_260 nm_ of 1.0 = 60 µg/ml RNP as conversion factor (12).

### Endonuclease assay

Endonuclease activity of the PA-Nter domain was measured in buffer containing 20 mM Hepes (pH 7.9), 100 mM sodium acetate, 1 mM Mn(OAc)_2_ and 2 mM DTT. We note here, as also previously reported (6), that the PA-Nter showed activity only in the presence of divalent ion Mn^2+^ (Supplementary Figure S2).

Unless mentioned otherwise, the RNP-based endonuclease and primer extension assays were performed in buffer containing 20 mM Hepes (pH 7.9), 100 mM sodium acetate, 1 mM Mg(OAc)_2_, 0.1 mM Mn(OAc)_2_, 10% glycerol and 2 mM DTT. The protein and RNA concentrations used in the assays are specified in the figure legends. Reactions were performed at 25°C. To generate radioactively labeled capped RNA, 5′ tri-phosphorylated RNA was capped at the 5′ end with α-P^32^-GTP using capping enzyme purchased from CellScript. Uncapped RNA was labeled at the 5′-terminus with γ-P^32^-ATP using T4 PNK purchased from Invitrogen. Following the endonuclease reactions, RNA products were separated in 20% polyacrylamide gels in 7 M urea and then visualized and quantified using a Molecular Dynamics PhosphorImager with ImageQuant software.

### Fluorescence assay

Fluorescence spectra were measured with a Jasco model 815 spectrophotometer equipped with fluorescence capabilities and temperature controller. The 2-AP modified RNA oligonucleotides were excited at 315 nm, and emission was recorded at 370 nm, at 25°C.

All the kinetic traces from both fluorescence and gel-based assays were fit to the following single exponential equation to obtain the apparent rate constant for the endonuclease reaction,
(1)
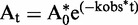

where A_0_ is the initial populations of substrate RNA, A_t_ is the population at time t, k_obs_ is the apparent rate constant, and t is time.

## RESULTS

### RNA cleavage site preference of the Influenza A RNA polymerase PA-Nter domain

**Figure 1.**
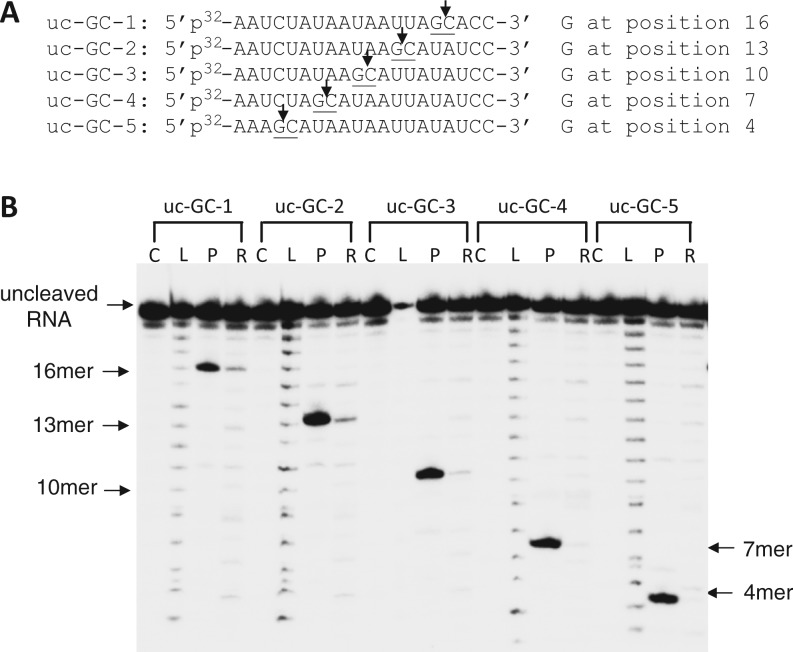
PA-Nter endonuclease exhibits sequence specificity of cleavage on uncapped RNA. (**A**) RNA sequences and their designations. (**B**) The cleavage pattern of the RNAs in the presence of PA-Nter domain and RNP. The 3 µM and 300 nM concentrations of RNAs were incubated with 1 µM and 8 nM PA-Nter domain and RNP, respectively, at 25°C for 1 h. Lanes were loaded with RNA alone (Lanes C), RNA Ladder after alkaline digestion (Lanes L), RNA incubated with PA-Nter (Lanes P) or incubated with influenza RNP (Lanes R). Total amount of RNA loaded in Lanes P were 10-fold lower relative to the amount loaded in Lanes R. The products of alkaline hydrolysis of the RNAs are used as markers.

Previous observations of endonuclease activity associated with native influenza RNP had indicated different cleavage efficiencies for different RNA substrates and in particular high-efficiency cleavage of substrates that carried GC motifs (10,13). To characterize the intrinsic selection of RNA cleavage sites by the influenza endonuclease, we first examined the ability of recombinant PA-Nter to cleave a variety of single-stranded uncapped RNAs (henceforth denoted as uc-RNA) that were radioactively labeled at the 5′ end. We used a collection of different 20-mer uc-RNAs, named GC-1, GC-2, GC-3, GC-4 and GC-5, each carrying a single 5′-AGCA-3′ motif at various distances from the 5′ end of the RNA (Figure 1A, Table 1). After incubation of these RNAs with PA-Nter (P) or native influenza RNP (R), the products of the endonuclease digestion were visualized after gel electrophoresis. The cleavage sites were determined by aligning with alkaline hydrolysis products (L) of the corresponding radioactively labeled substrate RNAs and by co-migration with synthesized marker RNA oligonucleotides of the same size and sequence as that of the cleaved products (Table 2). As shown in Figure 1B, PA-Nter (P) cleaved these RNA molecules highly selectively at the 3′ end of the G in each of these molecules. The position of the 5′-AGCA-3′ motif was different in each of the RNA substrates, and the cleavage site moved accordingly (Figure 1B).

**Figure 2.**
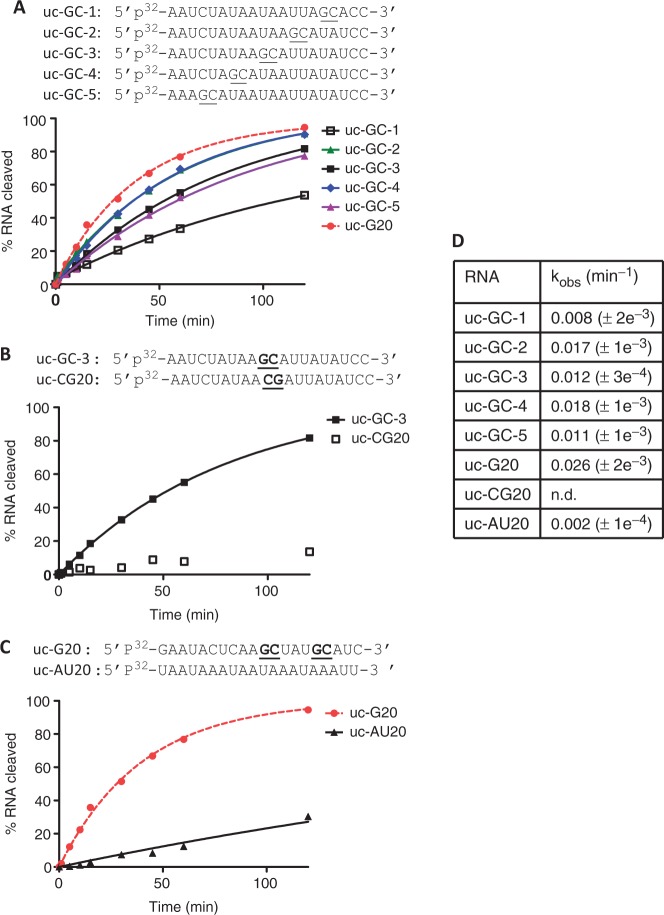
Kinetics of cleavage of various uncapped RNA sequences by PA-Nter domain. The sequences of RNAs are shown above the kinetic traces. Endonuclease reaction kinetics for (**A**) uc-GC1, uc-GC-2, uc-GC-3, uc-GC-4, uc-GC-5 and uc-G20; (**B**) uc-GC-3 and uc-CG20; (**C**) uc-G20 and uc-AU20 RNAs. The 3 µM RNA was incubated with 1 µM concentration of PA-Nter domain at 25°C. (**D**) The apparent rate constants (k_obs_) for the reactions are listed in the table.

**Figure 3.**
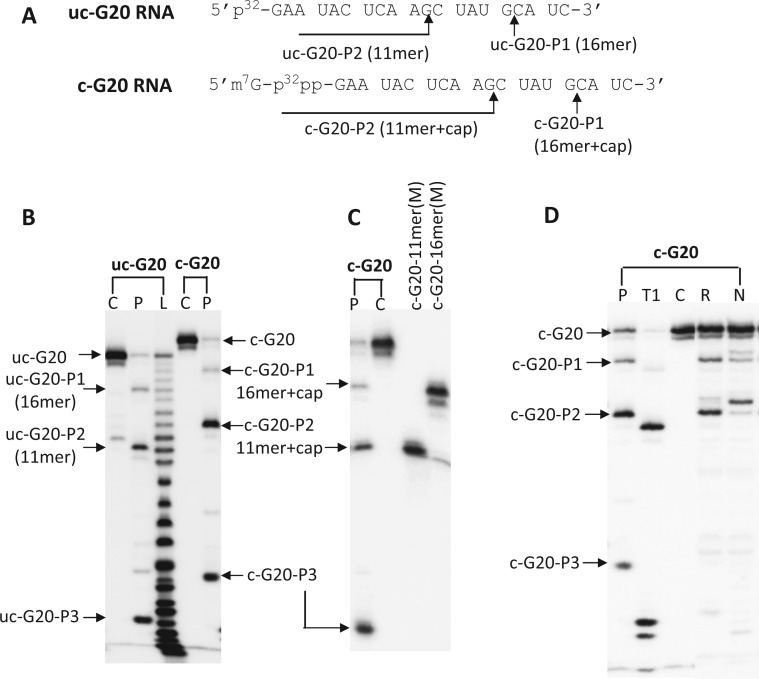
Denaturing gels showing the endonuclease digestion products from capped and uncapped G20 RNA after incubation with PA-Nter domain and RNP. (**A**) Sequence and nomenclature of the RNA substrates. The cleavage sites are marked with arrow. (**B**) Cleavage products from 300 nM capped or 3 µM uncapped G20 RNA after incubation with 1 uM PA-Nter. (**C**) Gel showing co-migration of c-G20-P1 and c-G20-P2 with respective synthesized marker RNA oligos c-G20-16-mer(M) and c-G20-11-mer(M). c-G20-16-mer(M) and c-G20-11-mer(M) are of the same size and sequence as that of c-G20-P1 and c-G20-P2, respectively (Table 2). (**D**) Endonuclease digestion of c-G20 RNA by PA-Nter, RNP and RNase T1. For all the gels, lanes were loaded with RNA alone (Lanes C), RNA Ladder after alkaline digestion (Lanes L), RNA incubated with PA-Nter (Lanes P) or incubated with influenza RNP (Lanes R), or with RNP and CTP (Lanes N) and RNA products from RNase T1 digestion (Lane T1). Incubation with CTP extended the RNA product by 1 base. RNase T1 cleaves at the 3′-end of G residues, leaving a 3′-phosphate group, while PA-Nter and RNP leave a 3′-OH group after cleavage.

To further characterize this unexpected cleavage site selectivity of PA-Nter, we examined three additional 20-mer uc-RNA sequences: (i) uc-G20, carrying two GC motifs 5′-AGCU-3′ and 5′-UGCA-3′, in a different sequence, (ii) uc-CG20, a derivative of the GC-3 oligonucleotide, in which the 5′–*GC*–3′ sequence was inverted to 5′-*CG*–3′; and (iii) uc-AU20 that lacks any G or C within the sequence (Table 1). Figure 2 shows the kinetics of the endonuclease reaction for these RNAs that were radioactively labeled at the 5′ end and visualized by gel electrophoresis (Supplementary Figure S1). PA-Nter generated three distinct products (uc-G20-P1, uc-G20-P2 and uc-G20-P3) from the uc-G20 substrate RNA (Supplementary Figure S1). The uc-G20-P1 and uc-G20-P2 corresponded to 16- and 11-mer, respectively, indicating endonuclease cleavage at the 3′ end of G of the two GC motifs. The shortest product, uc-G20-P3, migrated faster than the 2-nt marker RNA denoted as uc-G20-2mer(M) (Table 2, Supplementary Figure S1), consistent with cleavage after the 5′G (Supplementary Figure S1, bottom right panel and Figure 3B). As shown in Figure 2A, under comparable conditions, the rate of disappearance of full-length uc-G20 is the fastest relative to all the RNAs examined, consistent with the presence of three efficient cleavage sites in the uc-G20 sequence. As shown in Figure 2A, among the RNAs that contained a single efficient cleavage site, the distance of the G nucleotide from the 5′ end of the RNA appeared to have an effect on the rate of the reaction. Strikingly, the uc-CG20 substrate was not cleaved efficiently by PA-Nter (Figure 2B and Supplementary Figure S1). The cleavage of this substrate was considerably slower relative to uc-GC-3 (Figure 2B). The difference between these two substrates was only a switch of the 5′-GC-3′ motif to a 5′-CG-3′ motif at the same location in the RNA. This result suggests recognition of a directional positioning of GC for efficient cleavage by PA-Nter. Finally, we show that uc-AU20 serves as a poor substrate for the PA-Nter endonuclease as compared with RNAs containing GC motifs (Figure 2C). The aforementioned results therefore suggest that 5′-GC-3′ serves as the preferred RNA recognition motif for endonucleolytic cleavage by PA-Nter.

**Figure 4.**
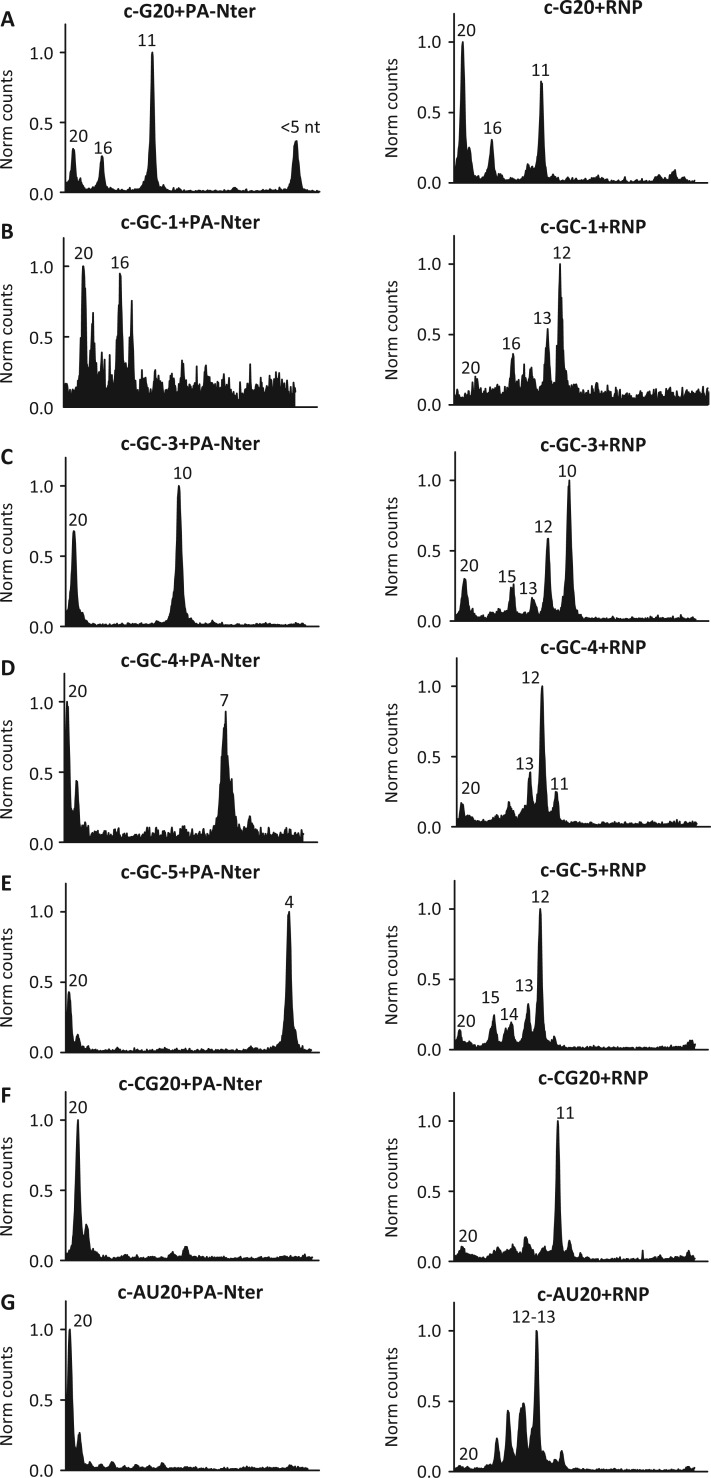
Cleavage pattern of PA-Nter and RNP on capped RNA substrates. The endonuclease reaction products were separated in a sequencing gel, and the gel lanes were scanned to generate the representative traces (gels shown in Supplementary Figure S3). The peaks correspond to the positions and intensities of the RNA species generated from the reactions. Sequence and nomenclature of the substrate and marker RNAs are shown in Tables 1 and 2. The size of cleavage products are denoted next to the peaks. The 300 nM concentrations of RNAs were incubated with either 1 µM PA-Nter domain (left panels) or 8 nM RNP (right panels) concentrations at 25°C for 1 h.

We also examined the effect of the presence of the m^7^GTP cap moiety (cap) at the 5′ end of the RNA (denoted as c-RNA) on cleavage by PA-Nter. Figure 3B and C show the cleavage products generated from c-G20 RNA. As, expected, this modified RNA substrate migrates slower compared with uc-RNA, owing to the presence of the cap. Like uc-RNA, PA-Nter domain generated three dominant products, denoted as c-G20-P1, c-G20-P2 and c-G20-P3, the sizes of c-G20-P1 and c-G20-P2 are (16 nt + cap) and (11 nt + cap), respectively, indicating cleavage at 3′ end of G of the GC motifs in the sequence (Figure 3C). The overall cleavage efficiency with PA-Nter was similar for capped and uncapped RNA. The exact size of c-G20-P3 could not be determined with certainty, but its small size suggests cleavage within 5 nt from the 5′ end of the capped substrate (Figure 3). Figure 4 shows the endonuclease cleavage reactions presented in the form of radioactive intensity traces of the polyacrylamide gels. The left panel of Figure 4 shows results from endonuclease reactions for a collection of related capped RNA molecules after incubation with PA-Nter. PA-Nter exhibited strong sequence preference and cleavage at the 3′ end of G for all 5′-GC-3′-containing RNAs examined (Figure 4 left panels A–E). In contrast, low activity and no sequence preference for cleavage was observed for RNA molecules that did not contain a 5′-GC-3′ motif (Figure 4, left panels F and G). Therefore, PA-Nter showed sequence selectivity for cleavage after a G residue in a 5′-GC-3′ motif in RNA with similar activity for capped and uncapped RNAs, consistent with the absence of a cap binding site on PA-Nter.

### Cleavage of capped and uncapped RNA by Influenza A RNP complex

Next, we determined the cleavage pattern for substrate RNAs by influenza A RNP complexes isolated from purified viruses. RNP complexes comprise the heterotrimeric influenza polymerase (PA, PB1, PB2) bound to the vRNA template that is coated with viral nucleoprotein (2,3). RNP-mediated endonuclease activity has been studied previously using labeled RNA substrates (12). Figure 1 shows the RNP-mediated cleavage of uncapped RNA molecules. Although cleavage efficiency was low for uncapped RNA, specific cleavage products were observed in these reactions. For uc-GC-1, uc-GC-2 and uc-GC-3 cleavage at the 3′ end of G generated the major reaction product, similar to the cleavage specificity observed with PA-Nter. Much lower activity was observed with uc-GC-4 and uc-GC-5 where the 5′GC3′ motifs are within 5 and 8 nt, respectively, from the 5′ end of the RNA.

Although the presence of cap did not affect the cleavage efficiency or RNA sequence specificity of PA-Nter (Figure 4, left panels), the endonuclease activity of influenza RNP was strongly increased by the addition of a cap structure and exhibited a distinctly different cleavage pattern as compared with uncapped RNA, whereas a preference for cleavage after G residues was maintained. Figure 4, right panels, show the RNP-mediated cleavage products from capped RNA substrates. The peaks represent the intensities of the RNA species generated from the endonuclease reactions and on visualization on sequencing gels (Supplementary Figure S3). RNP showed a cleavage preference for capped RNAs with a preferred distance of cleavage from the cap structure at ∼10–13 nt downstream from the 5′ cap, as reported previously (12). A careful inspection of the cleavage patterns for the various c-RNA sequences revealed that a dominating preference for guanine (G) was exhibited if present within the 10–13 nt distance range from the cap (for sequences c-G20, c-GC-3, c-CG20 RNAs). If that was not the case, the c-RNAs were cleaved with high selectivity at the 12th nt downstream from the cap (c-GC-1, c-GC-4, c-GC-5 and c-AU20 RNAs). Interestingly, unlike PA-Nter, RNP exhibited strong cleavage also at the 3′ end of the G for c-CG20 RNA sequence (Supplementary Figure S3). These results suggest that the influenza endonuclease cleavage specificity of RNP is determined by a combination of cap-binding-mediated distance measurement with an optimum at 12 nt from the 5′ end of the RNA and an intrinsic selectivity for cleavage after a G residue, which is conferred by the PA-Nter active site. As shown in Figure 3D, both RNP and PA-Nter generate the identical cleavage products c-G20-P1 (16-mer + cap) and c-G20-P2 (11-mer + cap) from c-G20 RNA, further confirming tha the observed sequence specificity of PA-Nter is also reflected in RNP.

**Figure 5.**
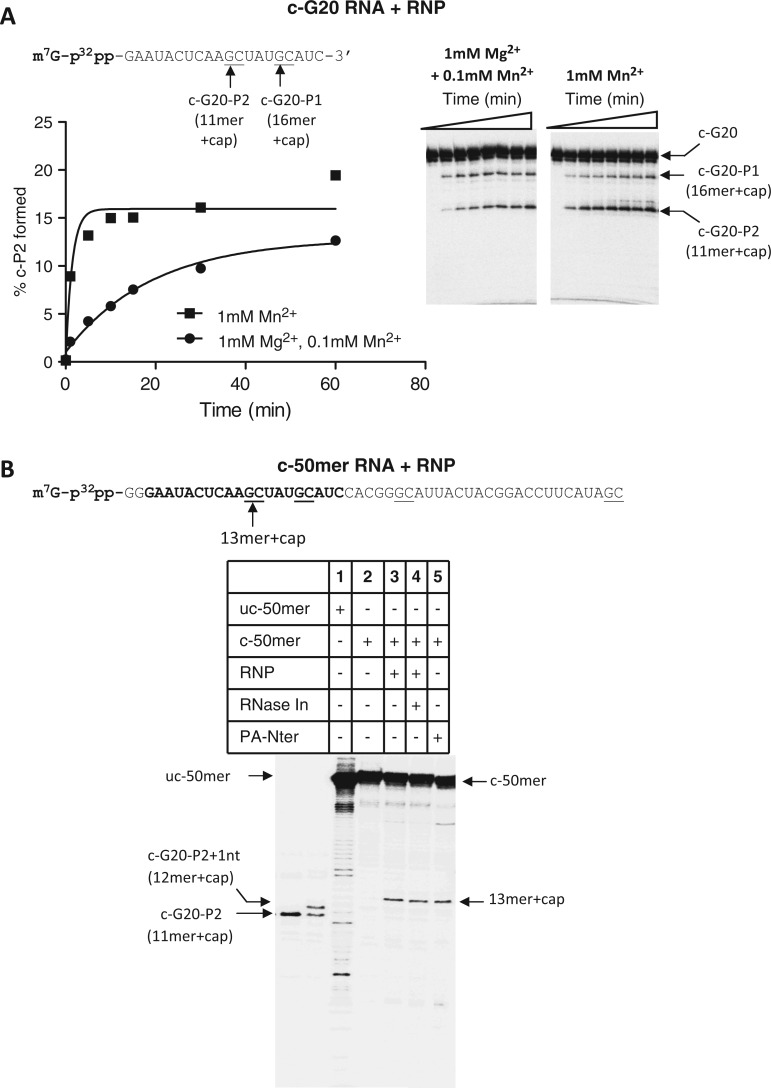
Endonuclease reaction products from capped G20 and 50-mer RNA by RNP and PA-Nter. (**A**) The effect of Mg^2+^ and Mn^2+^ on the cleavage pattern and reaction rate of RNP on c-G20 RNA. The concentrations of divalent metals used are stated within the figure. (**B**) Endonuclease products of 50-mer RNA in the presence of RNP and PA-Nter domain. c-G20-P2 and c-G20-P2 + 1 nt from RNP-mediated reactions were used as markers (left lanes). PA-Nter and RNP reactions were performed in buffers containing 1 mM Mn^2+^ and 1 mM Mg^2+ ^+ 0.1 mM Mn^2+^, respectively. The stretch of the 50-mer RNA that is identical to G20 sequence is highlighted in bold.

We note here that the divalent metal ion concentrations were different for the endonuclease reactions performed with PA-Nter (1 mM Mn^2+^) and RNP (1 mM Mg^2+^ and 0.1 mM Mn^2+^). PA-Nter did not show measurable endonuclease activity in the presence of 1 mM Mg^2+^ and 0.1 mM Mn^2+^ but required ≥1 mM Mn^2+^ concentration for detection of activity (Supplementary Figure S2). To test whether the presence of 1 mM Mn^2+^ might impact the cleavage pattern of capped RNA by RNP, RNP-mediated endonuclease reactions were also performed in 1 mM Mn^2+^. Figure 5A shows the endonuclease products generated by RNP from c-G20 RNA substrate in buffers containing either (1 mM Mn^2+^) or (1 mM Mg^2+^ and 0.1 mM Mn^2+^). As shown, for capped RNA, similar RNA products (c-G20-P1 and c-G20-P2) were generated by RNP in either buffer condition.

To examine whether the length of the RNA primer had any significant effect on the endonuclease cleavage pattern, we examined the cleavage characteristics on a longer 50-mer capped RNA substrate. This RNA was generated by T7 RNA polymerase-mediated transcription followed by capping; the sequence of this RNA is shown in Figure 5B. We note that the DNA template sequence for T7 transcription was designed to ensure that the 5′ stretch of the 50-mer RNA encoded by T7 RNA polymerase is identical to the G20 RNA sequence with two additional 5′ guanines as T7 RNA polymerase exhibits a marked preference for GTP as the initiating nucleotides (14). We observed that both RNP and PA-Nter cleaved 50-mer RNA predominantly at the 3′ end of the G situated at the 13th position downstream from the 5′ cap. Also, the cleavage pattern of the RNA by RNP was not affected by the presence of RNase Inhibitor (RiboGuard from Epicentre, WI) (Figure 5B, lane 4), indicating that the cleaved products resulted from intrinsic endonuclease activity of the PA endonuclease. The RNP-mediated cleavage product of c-G20 (c-G20-P2) and the single nucleotide extension product of c-G20-P2 in the presence of CTP (c-G20-P2 + 1 nt) were used as markers (left lanes) in Figure 5B.

### Kinetic analysis of RNA cleavage by PA-Nter using fluorescence based assay

We developed a fluorescence-based real time assay to determine kinetic parameters of the PA-Nter catalyzed endonuclease reaction. We used uc-G20 RNA with a 2-AP substitution as the model sequence, denoted as uc-G20-2AP (Table 1). The 2-AP is an analog of adenine, which exhibits characteristic fluorescence with excitation and emission maxima at 315 and 370 nm, respectively. The 2-AP-containing RNA has previously been used as a substrate in different polymerase and nuclease assays (15,16). When inserted into RNA or DNA sequence, the fluorescence intensity of 2-AP is significantly quenched due to stacking with adjacent bases, whereas the intensity is enhanced when the bases are unstacked (17). RNA cleavage near a 2-AP nucleotide is accompanied by a characteristic increase in fluorescence efficiency, which can be used as a marker for such a reaction. Here, we used this spectroscopic characteristic of 2-AP to monitor the kinetics of RNA cleavage by PA-Nter domain.

**Figure 6.**
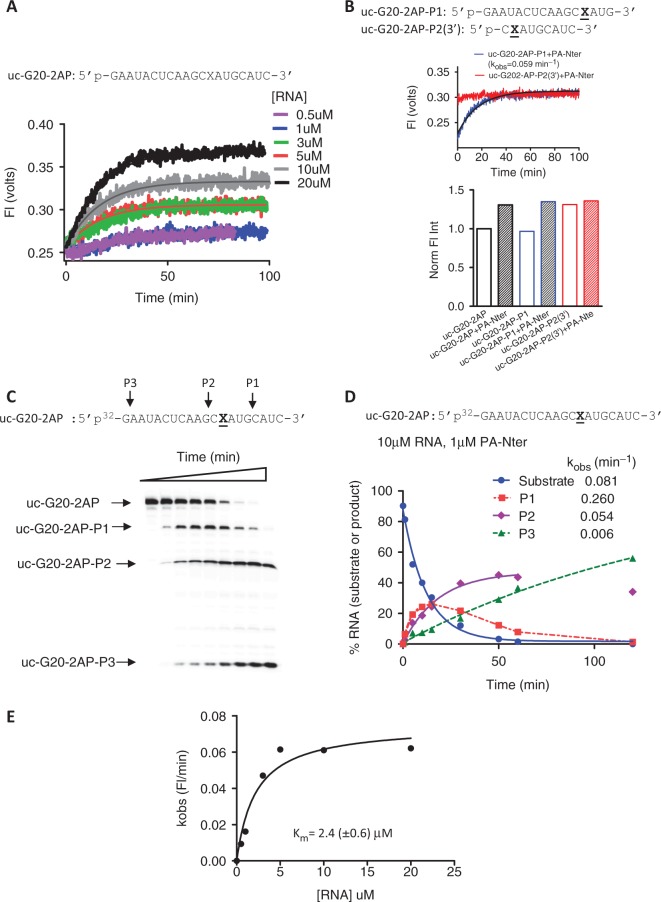
Fluorescence-based detection of PA-Nter endonuclease reaction kinetics using 2-AP modified uncapped G20 RNA substrate. The sequence and nomenclature of the RNAs used are shown above each trace. For (**B–D**), the RNA and protein concentrations were 10 and 1 µM, respectively. (**A**) Kinetic traces for PA-Nter domain at RNA concentrations specified in the figure. The starting fluorescence was normalized to the same value for all RNA concentrations for visual clarity. Concentration of PA-Nter was 1 µM. Each trace represents an average of two independent measurements. (B) Upper panel: the kinetic traces of the 2-AP-containing product RNAs in the presence of PA-Nter domain. Lower panel: histogram depicting the fluorescence of the substrate and product RNAs in the absence and presence of PA-Nter. The intensities were normalized relative to the intensity of the substrate RNA. (C) Sequencing gel showing the endonuclease products generated from the uc-G20-2AP RNA. (D) Kinetic analysis of the gel-based assay shown in C. (**E**) Michaelis–Menten fit of the k_obs_ values obtained from (A).

Figure 6A shows the kinetic traces of the endonuclease reactions at various RNA concentrations. As expected, 2-AP fluorescence intensity increased on incubation with PA-Nter under conditions that allowed RNA cleavage. To determine how the intensities of the cleaved products affect the overall change in the fluorescence intensity, we next measured the fluorescence of the RNA products that would likely contribute to the fluorescence change on cleavage. PA-Nter cleaved uc-G20-2AP substrate RNA into three distinct products (uc-G20-2AP-P1, uc-G20-2AP-P2 and uc-G20-2AP-P3) as observed for unmodified uc-G20 RNA, confirming that the presence of 2-AP did not affect cleavage site specificity of PA-Nter domain (Figure 6C).

Of the products, uc-G20-2AP-P1 and the 3′ end fragment of uc-G20-2AP-P2 [denoted here as uc-G20-2AP-P2 (3′)] are the most likely to contribute to the overall fluorescence change. We therefore measured the changes in intensities of these two RNAs in the absence and presence of PA-Nter domain (Figure 6B). Figure 6B, lower panel, shows that free uc-G20-2AP-P1 exhibited similar fluorescence as observed for the uc-G20-2AP RNA substrate. Therefore, this product did not contribute to the signal generated in the endonuclease reaction. The fluorescence intensity of the product RNA uc-G20-2AP-P2(3′) was 1.3× higher as compared withthe uc-G20-2AP RNA substrate. This agrees with the notion that 2-AP will be significantly less stacked in the P2 product as compared with the P1 product. The addition of PA-Nter to the uc-G20-2AP RNA substrate resulted in a 1.3× change in the fluorescence intensity, consistent with the generation of the uc-G20-2AP-P1 product. Similarly, when the reaction intermediate uc-G20-2AP-P1 was incubated with PA-Nter, there was a 1.3× change in fluorescence observed, whereas no further change in intensity was observed when uc-G20-2AP-P2(3′) was incubated with PA-Nter. In the absence of Mn^2+^, none of the aforementioned 2-AP-containing RNAs show change in fluorescence, indicating that Mn^2+^ is required for the cleavage activity of PA-Nter. Taken together, these results indicate that uc-G20-2AP-P2(3′) is the major fluorescent product species contributing to the observed change in fluorescence intensity during the endonuclease reaction, and that this assay can be used to monitor the rate of formation of this dominant species.

We next quantified and compared the rates of the endonuclease reactions from both the fluorescence and gel-based assays. The concentrations of RNA and PA-Nter domain used for the gel-based assay were 10 and 1 µM, respectively. All the kinetic traces from the fluorescence assay were fit to a single exponential to obtain the apparent rate constant for the endonuclease reaction [Equation (1)]. For the gel-based assay, the disappearance of the substrate RNA exhibited a single exponential behavior, whereas the dynamics of formation of the three products varied significantly (Figure 6D). The rate of formation of uc-G20-2AP-P1 was the fastest, which was converted to uc-G20-2AP-P2 and uc-G20-2AP-P3 over time. The traces were fit to single exponential (for uc-G20-2AP-P1 and uc-G20-2AP-P2, only the increasing phase was used for the fitting).

At comparable protein and RNA concentrations, the rate of formation of uc-G20-2AP-P2(3′) obtained from fluorescence assay (0.061 min^−^^1^) was similar to the rate of formation of uc-G20-2AP-P2 observed in gel-based measurements (0.054 min^−^^1^), and that these rates are in the same order of magnitude as the rate of overall disappearance of the substrate RNA was 0.081 min^−^^1^ in the gel-based assay (Figure 6D), showing that substrate disappearance is most directly correlated with the formation of P2. Taken together, these results demonstrate that the 2-AP-based assay can be used to obtain the kinetic parameters associated with the endonuclease cleavage of substrate uc-G20-2AP RNA.

The k_obs_ values obtained from the fluorescence assay were then plotted as a function of RNA concentration (Figure 6E), and the data were fit to Michaelis–Menten equation to obtain the apparent K_m_ for RNA of 2.4 µM for the reaction.

## DISCUSSION

It has previously been described that influenza RNA polymerase in the context of the viral RNP shows characteristic non-random cleavage of host capped mRNA substrates, which then serve as primers for initiation of viral transcription, a process also known as ‘cap-snatching’. The host mRNA cleavage appeared largely to be determined by the physical separation and distance between the cap-binding site in PB2 subunit and the endonuclease active site in PA subunit of viral RdRP (12). In this study, we show that influenza RdRP endonuclease exhibits a preference to cleave after guanine (G) in the mRNA leader sequence during cap snatching. Our study further shows that for mRNAs lacking G between 10 and 13 nt from the 5′ end of the cap, influenza RNP complex cleaves the leader precisely at the 12th nt, whereas in the presence of G in this region, G specificity of cleavage dominated over the position specific incision. The biochemical data presented here revealed that this sequence selectivity of the influenza RNP is intrinsic to the PA endonuclease active site of the RdRP, which is independent of the presence of viral template RNA. Our results show that isolated PA-Nter exhibits an intrinsic specificity to cleave at the 3′ end of G and also required the presence of an adjacent 3′-C. Taken together, this study provides the first direct biochemical evidence that the PA-Nter possesses G-specific mRNA cleavage activity, and that this characteristic is also exhibited by the polymerase in the RNP complex purified from influenza virus.

Consistent with our aforementioned observations, a recent *in vitro* competition study in which 2, 4 or even up to 11 capped leaders were offered indicated that 5′-GC-3′ is probably the preferred site of cleavage in the leader mRNA by influenza polymerase (18), which contradicted the previously reported observation that in infected cells, cleavage of donor pre-mRNA preferentially occurred after 5′-CA-3′ (19). Sequencing of influenza mRNAs have suggested an almost exclusive presence of a G residue corresponding to the conserved C residue at the second position of the viral template vRNA, whereas the nature of the nucleotide before the G could differ (18,20–22). In a majority of cases, the nucleotide before G was found to be an A, although other nucleotides were also observed. This was interpreted to indicate that the influenza endonuclease preferentially cleaved at the 3′end of an A residue and that a GTP was used as an initiating nucleoside triphosphate by the influenza polymerase. However, all available data are also consistent with a preferential endonuclease cleavage after a G residue and a preferential initiation of transcription with CTP as the initiating nucleoside triphosphate. It has been described that influenza polymerase could initiate transcription *in vitro* with GTP or CTP as a substrate (23). However, initiation with GTP tended to induce primer slippage and incorporation of more than one G residue, whereas initiation with CTP tended to result in single-defined initiation products. Also, capped alpha globin mRNA, which was preferentially cleaved to form a capped RNA primer ending in a GG dinucleotide, was not used for initiation with GTP, but only with CTP. Taken together, all available data are consistent with preferential capped RNA cleavage at the 3′-end of a G residue by the influenza endonuclease and transcription initiation with CTP. This striking preference for cleavage after a G residue is contributed as an intrinsic feature by the architecture of the PA subunit. It will be of high interest to determine the structure of the PA protein in complex with RNA substrate to determine the molecular nature of this base recognition.

Recently high-resolution co-crystal structures of PA-Nter with rUMP and dTMP (9) and rUMP, rAMP and TMP (24) have been reported, whereas attempts to co-crystallize the protein with any of the other d/rNMPs were unsuccessful. Although these studies provide useful information on how the ribonucleobases might interact within the active site, the extrapolation towards binding of oligonucleotide is difficult from these structures. High-resolution structures of the protein with substrate mRNA of various RNA sequences will be required to investigate the structural features of the PA active site that result in the GC motif selectivity of cleavage.

Sequence analysis of the 5′ cap-proximal region of viral mRNA from other negative strand RNA viruses had previously indicated nucleotide preference at the 3′ end of the capped primer (25–28). Based on these observations, it appears that the observed sequence preference results in the use of a specific subset of host cell capped mRNAs to generate primers for transcription of viral mRNA. Recent observations from *in vitro* studies have suggested that the polymerase selects host mRNA with base complementarity to the 3′ nucleotides of the vRNA template, which for influenza A are 3′-UCGUUUU … 5′ (18). The consensus sequence that has been reported for a preferred capped RNA leader is m^7^G-(N)_7-8 _-(A/U/G)-(A/U)-AGC-3′ (18), consistent with the preferred usage of GC terminated host mRNA leader. Nonetheless, as we have shown here and also previously reported (29), non-GC containing mRNA can also serve as primers for cap snatching. It might also be possible that the virus uses selected host mRNA sequences as primers in an attempt to inhibit the expression of cellular genes that are associated with the host antiviral response. More extensive studies involving deep sequencing of viral mRNA from infected cells would be required to establish the molecular significance of the sequence specificity of influenza endonuclease.

Here, we have also described a spectroscopic assay to elucidate the kinetics of RNA cleavage by PA-Nter of influenza endonuclease. Time-dependent changes in fluorescence intensity of 2-AP modified RNA oligos were measured to estimate the endonuclease reaction rates. We show that the kinetic parameters obtained from this method are consistent with the data from gel-based assay, confirming the validity of this assay. We further demonstrate that the catalytic efficiency of PA-Nter is dependent on the RNA sequence where GC containing RNA was cleaved with maximal efficiency while the activity was minimal for AU rich sequences.

Based on the aforementioned findings, we conclude that influenza endonuclease exhibits strong sequence dependence of RNA cleavage, which is an intrinsic property of the PA endonuclease active site. Further *in vitro* and *in vivo* studies will be required to fully understand the biological significance of such mRNA leader sequence preference in the lifecycle of the virus.

## SUPPLEMENTARY DATA

Supplementary Data are available at NAR Online.

## FUNDING

Hoffmann-La Roche, Inc. (to K.D. and K.K.); European Union Seventh Framework Programme (FP7) project FLUPHARM [259751 to A.W., O.S. and S.C.]. Funding for open access charge: Hoffmann-La Roche Inc., Nutley, NJ.

*Conflict of interest statement.* None declared.

## Supplementary Material

Supplementary Data
