# Supplementary material for: Characterization of PA-N terminal domain of Influenza A polymerase reveals sequence specific RNA cleavage
Source: Nucleic Acids Res. 2013 Jul 11;41(17):8289–99. doi: 10.1093/nar/gkt603 (PMC3783182; doi:10.1093/nar/gkt603)
Supplement: Supplementary Data [file supp_41_17_8289__index.html]

Characterization of PA-N terminal domain of Influenza A polymerase reveals sequence specific RNA cleavage — Characterization of PA-N terminal domain of Influenza A polymerase reveals sequence specific RNA cleavage — Supplementary Data 

# Characterization of PA-N terminal domain of Influenza A polymerase reveals sequence specific RNA cleavage

## 

files

**Files in this Data Supplement:**

- Supplementary Data - pdf file
