# Supplementary material for: Characterization of PA-N terminal domain of Influenza A polymerase reveals sequence specific RNA cleavage
Source: Nucleic Acids Res. 2013 Jul 11;41(17):8289–99. doi: 10.1093/nar/gkt603 (PMC3783182; doi:10.1093/nar/gkt603)

Figure S-1. Denaturing urea gels showing the kinetics of uncapped RNA cleavage by recombinant influenza PA-Nter protein. Sequences of the substrate RNA oligos are listed in Table 1. The kinetic analyses are shown in Fig. 2. The RNA size markers (denoted by the size of the marker followed by (M)) were synthesized RNA oligos carrying the same sequence as the corresponding product RNA. The sequences of the marker RNAs are shown in **Table 1B.**

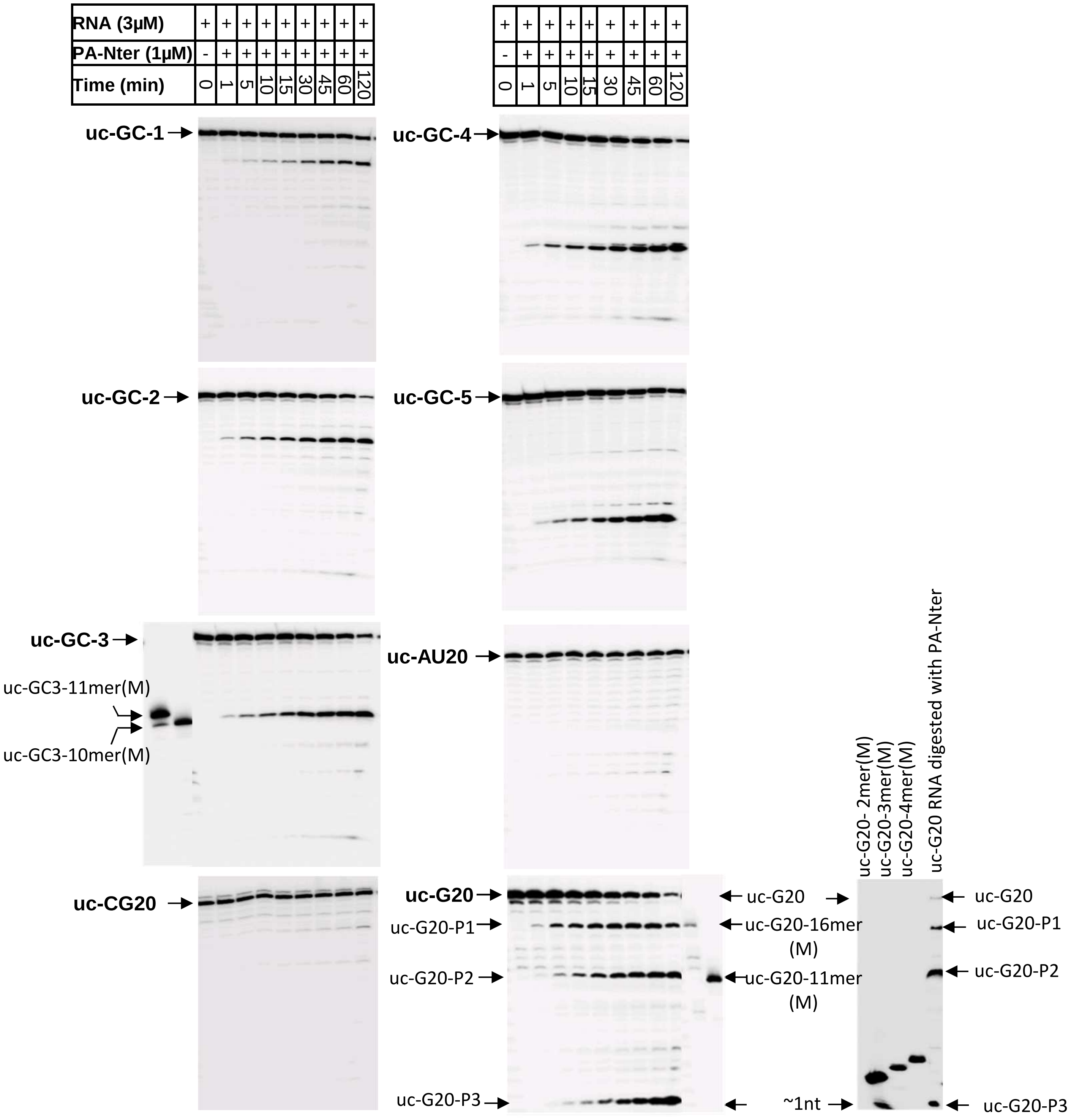

Figure S-2. Endonuclease activity of PA-Nter in various divalent metal ion conditions. 300nM concentration of c-G20 RNA was incubated with 1µM PA-Nter at 25°C for up to 2 hours either in the absence of any divalent ions or in the presence of 1mM Mn^{2+} , 1mM Mg^{2+}, 1mM Mg^{2+} + 0.1mM Mn^{2+} or 1mM Ca^{2+}.

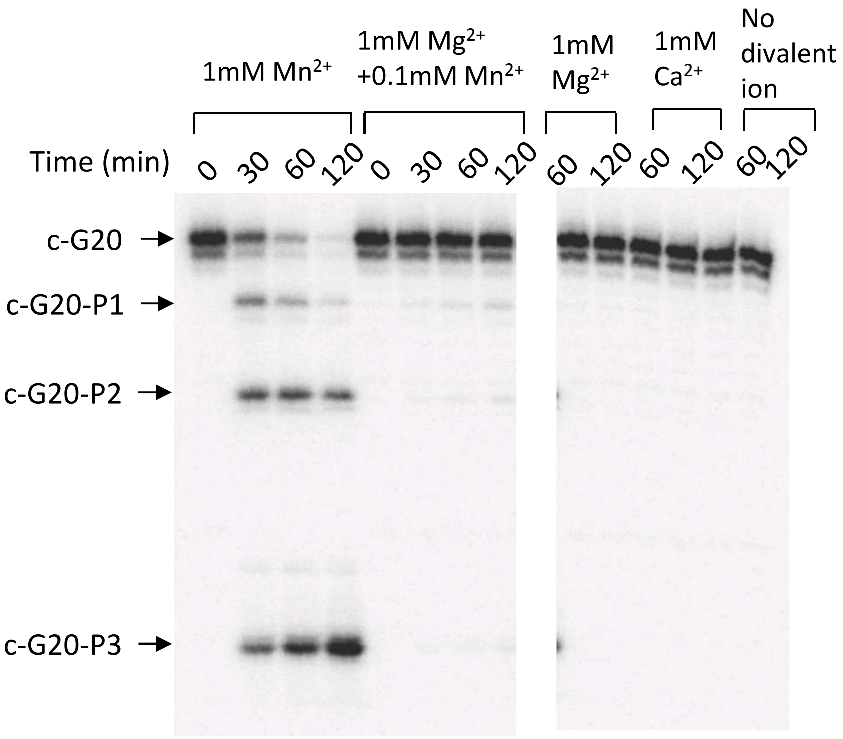

Figure S-3. Cleavage of capped RNA by RNP is dominated by cap binding. Denaturing gels showing the cleavage pattern of the RNAs in the presence of PA-Nter domain and RNP. RNA sequences for substrates and markers and their designations are shown in Table 1. Lanes were loaded with RNA alone (Lanes C), RNA incubated with PA-Nter (Lanes P) or incubated with influenza RNP (Lanes R) and RNA incubated with RNP and CTP (Lanes N). 300nM concentrations of RNAs were incubated with either 1µM PA-Nter domain or 8nM RNP concentrations at 25°C for 1 hr.

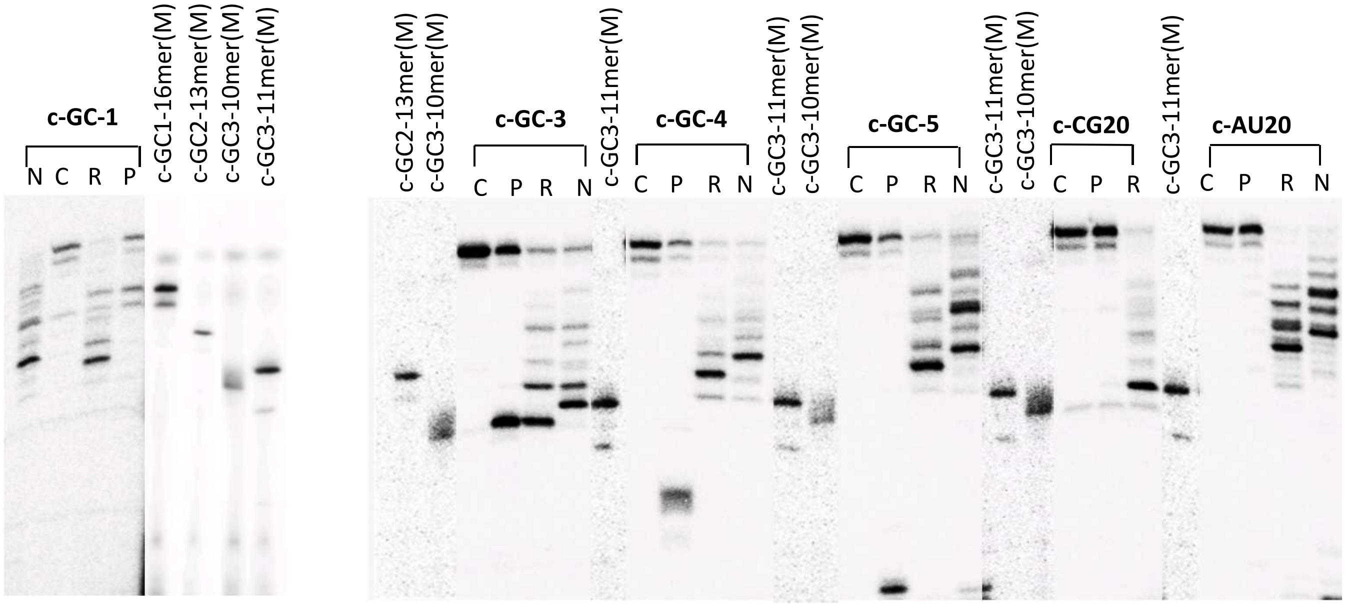

Supplement: Supplementary Data [file supp_gkt603_nar-01324-r-2013-File003.pdf]
